# First description of the male of *Cyclocosmiaricketti* (Araneae, Halonoproctidae) from China

**DOI:** 10.3897/BDJ.10.e79205

**Published:** 2022-01-20

**Authors:** Ye-Jie Lin, Linrui Yu, Xunyou Yan, Shuqiang Li

**Affiliations:** 1 Hebei Key Laboratory of Animal Diversity, College of Life Science, Langfang, China Hebei Key Laboratory of Animal Diversity, College of Life Science Langfang China; 2 Jinshan College of Fujian Agriculture and Forestry University, Fuzhou, China Jinshan College of Fujian Agriculture and Forestry University Fuzhou China; 3 Institute of Zoology, Chinese Academy of Sciences, Beijing, China Institute of Zoology, Chinese Academy of Sciences Beijing China

**Keywords:** Fujian, sclerotised disc, taxonomy, trapdoor spider

## Abstract

**Background:**

The genus *Cyclocosmia* Ausserer, 1871 of the spider family Halonoproctidae Pocock, 1901 includes ten known species from North America and Asia. Since *Cyclocosmiaricketti* was described by Pocock in 1901, no males of this species have ever been reported.

**New information:**

The male of *Cyclocosmiaricketti* is described for the first time, based on a specimen collected near the type locality in Fujian Province, China. A morphological description and illustrations are given.

## Introduction

The genus *Cyclocosmia* Ausserer, 1871 of the spider family Halonoproctidae Pocock, 1901 includes ten known species from North America (3) and Asia (7), six of them being found in China: *C.lannaensis* Schwendinger, 2005 (♂♀, China, Thailand), *C.latusicosta* Zhu et al., 2006 (♂♀, China, Vietnam), *C.liui* Xu et al., 2017 (♀, China), *C.ricketti* (Pocock, 1901) (♀, China), *C.sublatusicosta* Yu & Zhang, 2018 (♂, China) and *C.subricketti* Yu & Zhang, 2018 (♂♀, China) (World Spider Catalog 2021; Li 2020).

*Cyclocosmiaricketti* was initially reported as *Halonoproctusricketti*, based on one female specimen from Kuatun, north-western Fokien in China, now known as Guadun (27.7359°N, 117.6408°E) in Fujian Province. A *Cyclocosmia* male was collected 130 km (in a straight line) away from the type locality, which we believe to be conspecific and which we describe here (Fig. 6)

## Materials and methods

The specimen was preserved in 80% ethanol and examined under a LEICA M205C stereomicroscope. Images were taken with an Olympus C7070 zoom digital camera (7.1 megapixels). Habitus photographs of the preserved specimen were taken with a Sony A7RIV digital camera, equipped with a Sony FE 90mm Goss lens. Photos were stacked with Helicon Focus (Version 7.6.1) and processed with Adobe Photoshop CC2019.

All measurements are in millimetres and were obtained with an Olympus SZX16 stereomicroscope with a Zongyuan CCD industrial camera. The total length does not include the chelicerae. Eye sizes are measured as the maximum diameter in either dorsal or frontal view. Leg measurements are given as follows: total length (femur, patella, tibia, metatarsus, tarsus).

Specimens, reported here, are deposited in the Institute of Zoology, Chinese Academy of Sciences (IZCAS) in Beijing and Muséum d'histoire naturelle (MHN) in Geneva.

Abbreviations: **AE** apophysis of embolic tip, **ALE** anterior lateral eyes, **AME** anterior median eyes, **E** embolus, **PLE** posterior lateral eyes, **PME** posterior median eyes.

## Taxon treatments

### Cyclocosmia ricketti

(Pocock, 1901)

CB4E14C0-5F5E-533B-8CDB-DF81A57D9121

https://www.gbif.org/species/2163801

Halonoproctus ricketti Pocock, 1901 in Pocock (1901): 209, pl. 21, fig. 1.
Cyclocosmia ricketti in Simon (1903): 885, figs. 1044–1047; Gertsch and Platnick (1975): 18,19, figs. 28, 29, 32 and 36 (part); Song et al. (1999): 36, figs. 16H and K–L (part); Schwendinger (2005): 227, figs. 2–8, pl. 1D; Zhu et al. (2006): 120, figs. 1 and 2; Zhang et al. (2007): 385, fig. 101; Yin et al. (2012): 134, figs. 13a–e; Xu et al. (2017): 78, figs. 1B–L.

#### Materials

- **Type status:** Other material. **Occurrence:** catalogNumber: IZCAS-Ar41617; occurrenceRemarks: found in gutter; recordedBy: Linrui Yu; individualCount: 1; sex: male; lifeStage: adult; **Location:** continent: Asia; country: China; countryCode: China/CN; stateProvince: Fujian; verbatimLocality: Nanping City, Yanping District, Mangdang Mountain, near Hengkeng; verbatimElevation: 1078–1083 m; verbatimLatitude: 26.6395°N; verbatimLongitude: 118.0777°E; **Event:** eventTime: GMT+8 22:30; year: 2021; month: 5; day: 2

#### Description

Male: Body length 13.41. Carapace 6.19 long, 5.61 wide, dark reddish-brown, slightly curved retrolaterally and rounded posteriorly, most of clypeal area covered with reticular fuscous veins, thin ridges transversely running across area from clypeus to fovea. Clypeus height 1.32; Few short, firm bristles concomitantly with ridges in front of eyes and sparsely dispersed on and behind eye formation; no thick long bristles discernible.

Eyes (Fig. 1B) situated on low mound and far back from anterior margin of carapace, eye arrangement as in female, all eyes bright off-white, eye group 1.64 long, 0.90 wide. Eye diameters and interdistances: AME 0.39, ALE 0.38, PME 0.27, PLE 0.30; AME-AME 0.15, AME-ALE 0.16, AME-PME 0.16, PME-PME 0.68, PME-PLE 0.05, ALE-PLE 0.13; fovea similar to that of female, occupying about one fourth of carapace width at that point.

Chelicerae relatively slender, similar to carapace in pigmentation, retrolaterally with dark reticular pattern and thin wrinkles; promargin of groove with seven principal teeth and five denticles, retromargin with ten sizable denticles, arranged in regular rows; dense hair covering retromargin; prolateral surface of fang with serrated longitudinal keel; rastellum conspicuously projected, with about eight distal spines.

Maxillae 4.07 long, 2.49 wide, yellow-brown, with about five tiny cuspules at prolateral corner of ventral surface and numerous white setae on proventral surface.

Labium (Fig. 1C) 0.86 long, 1.29 wide, with same pigmentation as maxillae, tip with few gracile hairs.

Sternum (Fig. 1C) 3.53 long, 3.56 wide, light yellow-brown, with paramedian inconspicuous flower-shaped sigillum in its centre.

Palp (Fig. 2 - showing palp with distorted palpal organ) with smooth surface, femur almost glabrous and ornamented with numerous transversal striae; distal part of patella bearing few scattered black hairs; tibia cylindrical, basal half slightly inflated and distal part narrow, ventrally covered with hairs; tarsus short, dorsally with several hairs, with vaulted emarginate apex; width of bulbous part of palpal organ in retrolateral view 1.33 (Fig. 4), divided into left and right parts by deep angular distal invagination (Fig. 3A); embolus linear in ventral and dorsal view, slightly curved in lateral view, its apex tapering, length about 2.39 (Fig. 4); apophysis of embolic apex pointing dorsad, with tip of embolus narrowly rounded (Fig. 5d).

Legs distinctly more slender and smoother than in female, light reddish-brown, with conspicuous longitudinal bands on dorsal surface and fine transversal fissures; tarsi, metatarsi and tibiae armed with blunt setae and black spines scattered on ventral side; ventral side of all tarsi, except tarsus IV, with dense short, white scopula covering about distal half of the segment. Legs I and II with setae and spines denser than on legs III and IV; patellae and femora almost glabrous; two spines inside tarsal scopula on leg I, three spines on leg II, five spines on leg III, seven spines on leg IV; few short spines present laterally on dorsal side of all patellae; distal part of tarsus IV ventrally with a few gracile, serrate white hairs. Leg measurements: I 17.53 (5.63 + 6.07 + 3.86 + 1.97), II 14.62 (4.56 + 4.63 + 3.34 + 2.09), III 14.08 (4.15 + 4.57 + 3.11 + 2.25) and IV 17.93 (5.09 + 5.72 + 4.51 + 2.61). Leg formula (from longest to shortest leg): 4123.

Trichobothria tibia, metatarsus and tarsus surface with few, terminal one longest.

Paired tarsal claws with two teeth near the base; unpaired claws bare.

Abdomen (Fig. 1D) 7.22 long, its rounded disc 5.17 in diameter, dark yellow-brown, clearly less sclerotised than in female; tergum with few adnate blunt bristles and an conspicuous cardiac mark mid-dorsally; abdominal disc with narrow ribs and shallow grooves, 30 radiating ribs on each side; margins of abdominal seam inconspicuous, rib angles slightly elevated, each with several small hairs; positions and shapes of muscle impressions as in female, but only two pairs of short bristles on their rims.

Spinnerets: posterior median spinnerets thin, one-segmented, 0.71 long; posterior lateral spinnerets relatively thick, three-segmented, 1.51 long (proximal segment 0.57, median 0.36, distal 0.57) with with distal segment narrower than others; anus covered by crescent-shaped sclerite, not connected to ventral median rib angle (Fig. 1E).

##### Comparative material studied

*Cyclocosmialannaensis*, 1♂ (MHN, Fig. 5d), CHINA, Yunnan Province, Menglun, 16.–31.V.2007; *Cyclocosmialatusicosta*, 2♂ (IZCAS, Fig. 5a), VIETNAM, Ninh Binh Province, Cuc Phuong National Park, pitfall traps, 1.–30.I.2008, leg. Pham Dinh Sac; *Cyclocosmiasiamensis*, 1♂ (MHN, Fig. 5b), THAILAND, Doi Suthep.

#### Diagnosis

*Cyclocosmiaricketti* differs from other species of *Cyclocosmia* by the character of 23–33 radiating ribs on each side of abdominal disc (Fig. 1D) (vs. 20–23 in *C.lannaensis* and *C.latusicosta*, 29–33 in *C.siamensis*, 34 in *C.sublatusicosta* and 32–34 in *C.subricketti*). It can be distinguished from *C.latusicosta* by the lack of the elevated central zone inside the upper pair of muscle impressions (Fig. 1D) (vs. elevated central zone present in *C.latusicosta*) and it differs from *C.siamensis*, *C.subricketti* and *C.sublatusicosta* by the latter in the upper and median pair of muscle impressions on the opisthosomal disc separated by one transversal rib (vs. separated by two transversal ribs in *C.siamensis*, *C.subricketti* and *C.sublatusicosta* (Schwendinger 2005 Fig. 22; Yu and Zhang 2018 Figs. 4A and 5G)).

The male of *C.ricketti* can be distinguished by the following characters: in the ventral view of the palp organ, the diameter of the bulb is about 1/2 of the embolus length (vs. 1/3 in *C.latusicosta* and *C.sublatusicosta* (Yu and Zhang 2018 Figs. 1D and 3D)), both sides of the bulb are separated by an angular invagination in ventral view (Fig. 4A and B) (vs. rounded invagination in *C.lannaensis* (Schwendinger 2005 Figs. 41–44)) and in prolateral view, the apophysis of the embolic tip points dorsally (vs. points laterally in *C.latusicosta*, *C.siamensis* and *C.lannaensis*) (Fig. 5).

In females, the length of spermathecae to its width is 3:2 (Xu et al. 2017 Figs. 1G and H; Zhu et al. 2006 Fig. 2E) (vs. 2:1 in *C.latusicosta*, *C.liui and C.siamensis* (Xu et al. 2017 Fig. 3F; Zhu et al. 2006 Figs. 6F–J; Schwendinger 2005 Figs. 17–21)).

#### Distribution

China (Fujian, Hunan, Jiangxi, Sichuan, Zhejiang) (Fig. 6)

## Supplementary Material

XML Treatment for Cyclocosmia ricketti

## Figures and Tables

**Figure 1.**
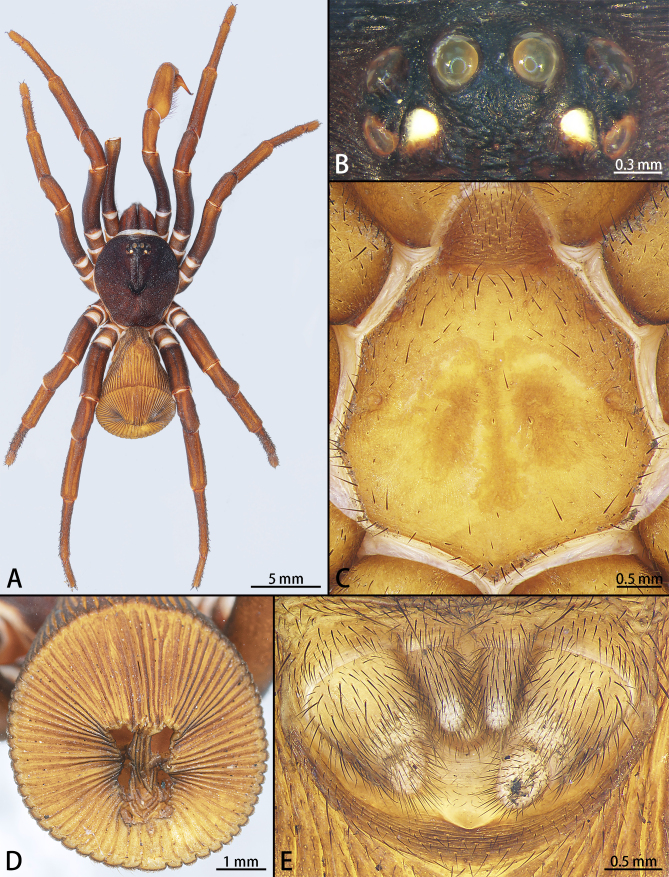
*Cyclocosmiaricketti*, male from Mangdang Mountain: **A** habitus, dorsal view; **B** eye group; **C** labium and sternum, ventral view; **D** abdomen, posterior view; **E** spinnerets, ventral view.

**Figure 2.**
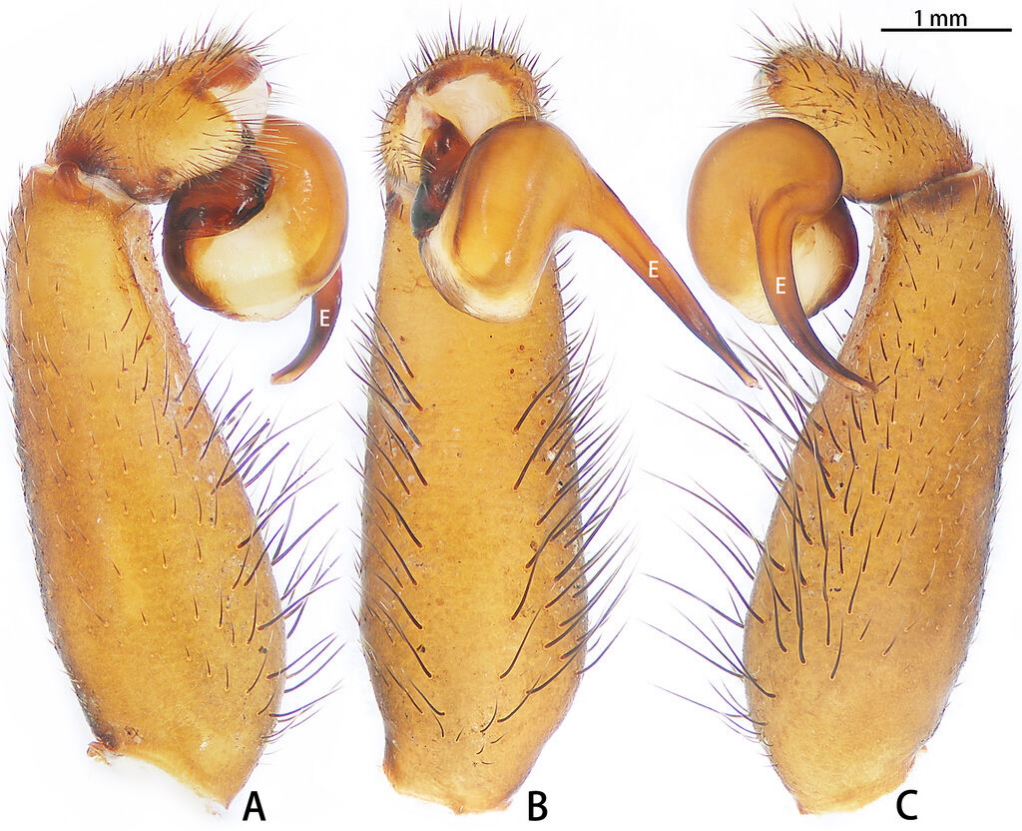
*Cyclocosmiaricketti*, male from Mangdang Mountain, left palp with distorted palpal organ: **A** prolateral view; **B** ventral view; **C** retrolateral view.

**Figure 3.**
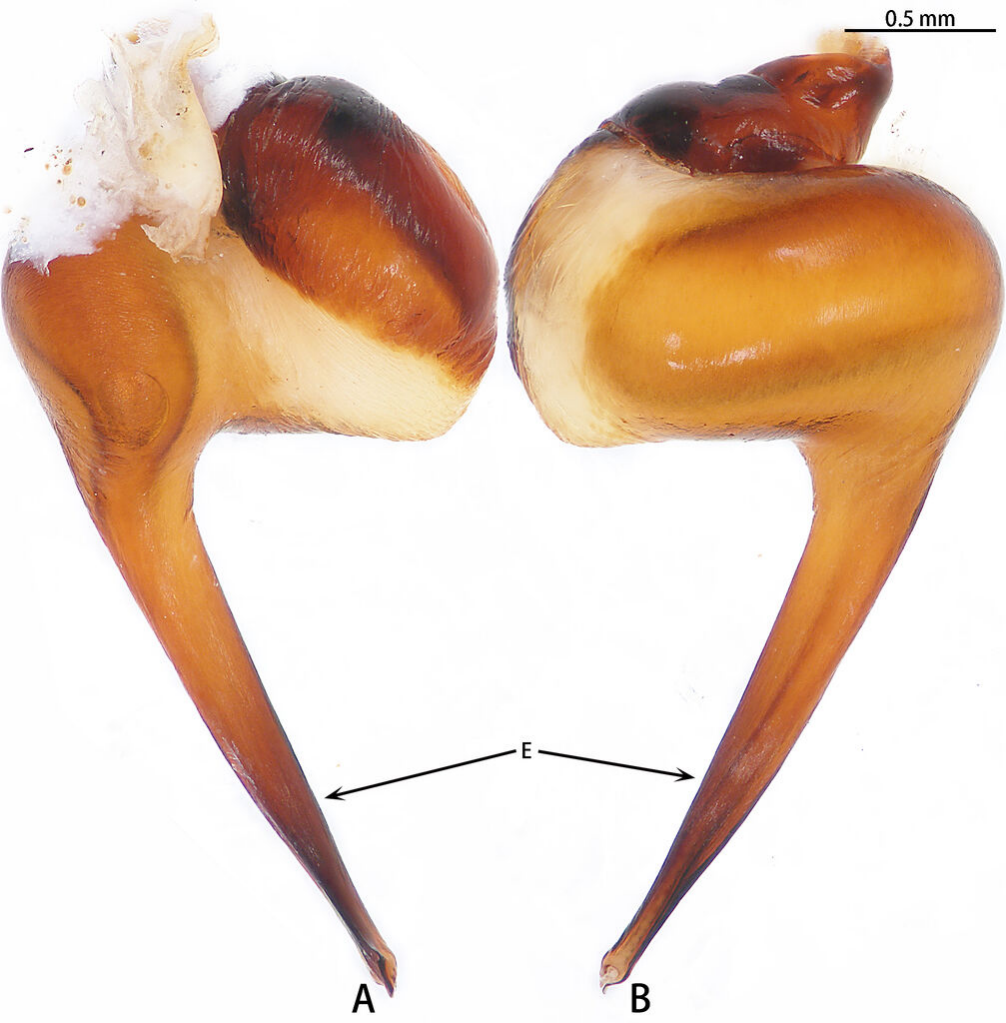
*Cyclocosmiaricketti*, male from Mangdang Mountain, right palpal bulb organ: **A** ventral view; **B** dorsal view.

**Figure 4.**
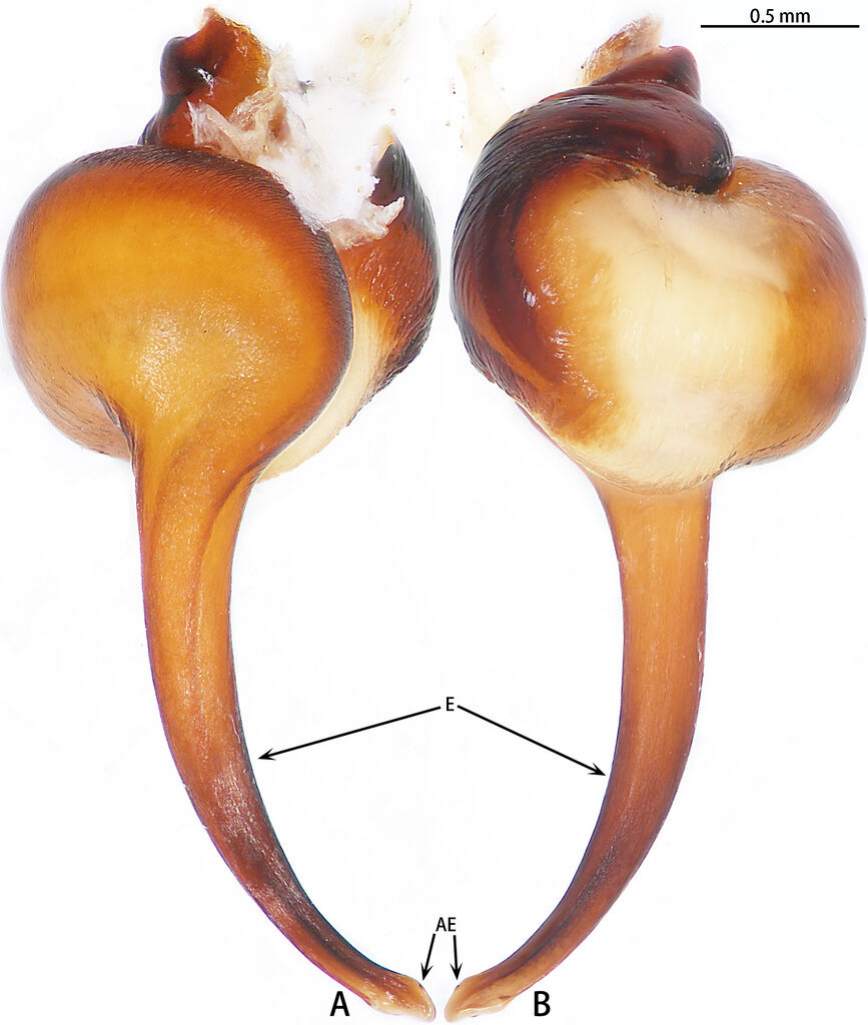
*Cyclocosmiaricketti*, male from Mangdang Mountain, right palpal bulb organ: **A** prolateral view; **B** retrolateral view.

**Figure 5a.**
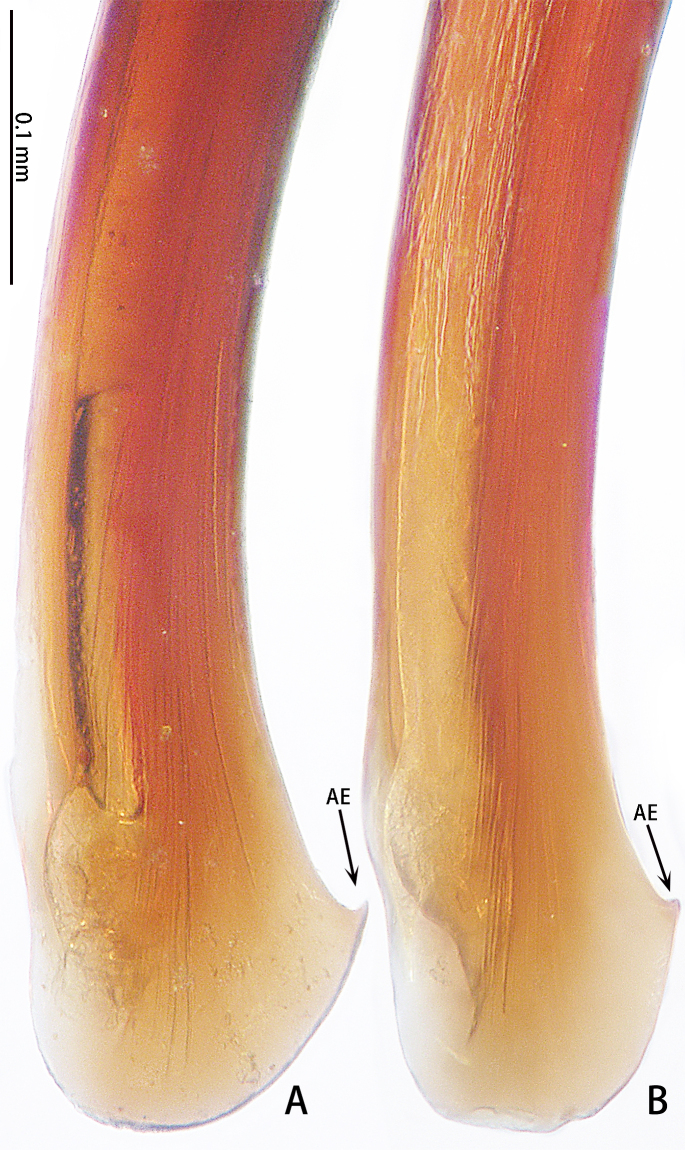


**Figure 5b.**
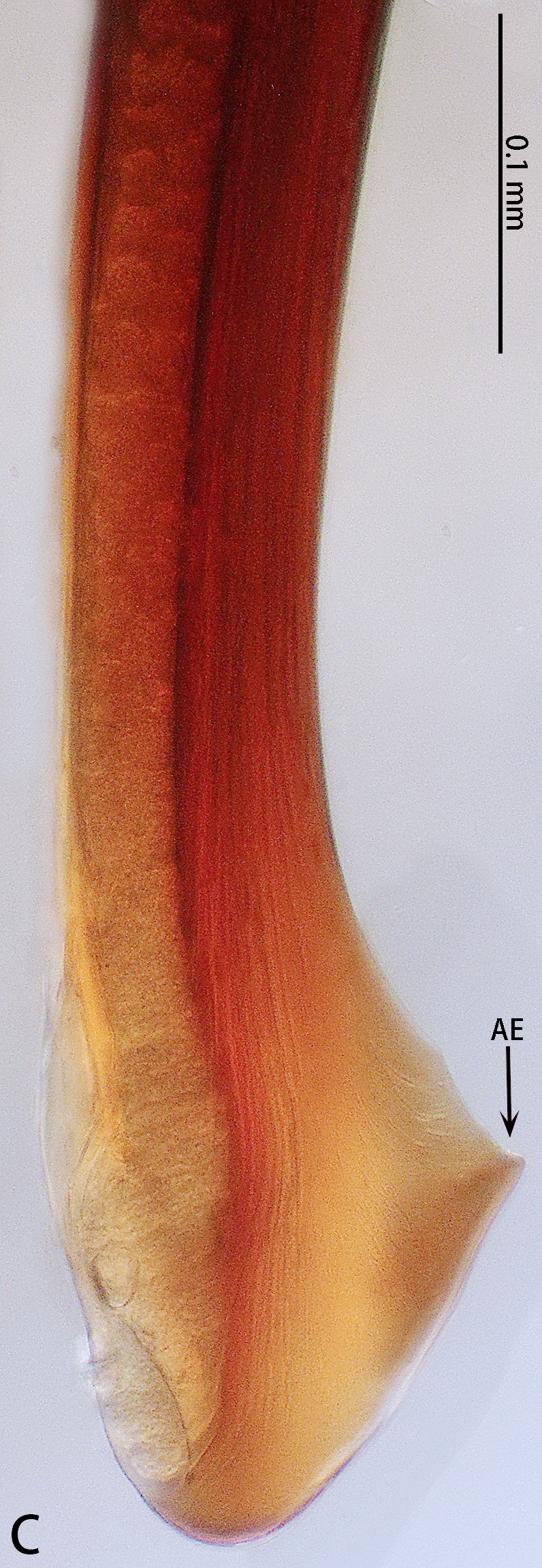


**Figure 5c.**
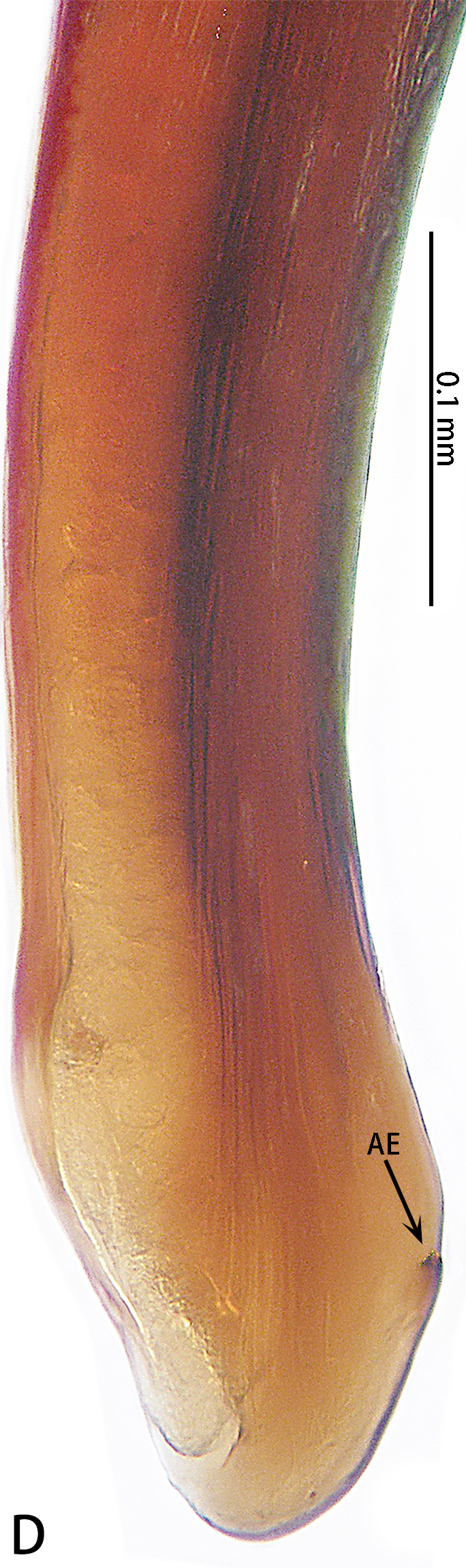


**Figure 5d.**
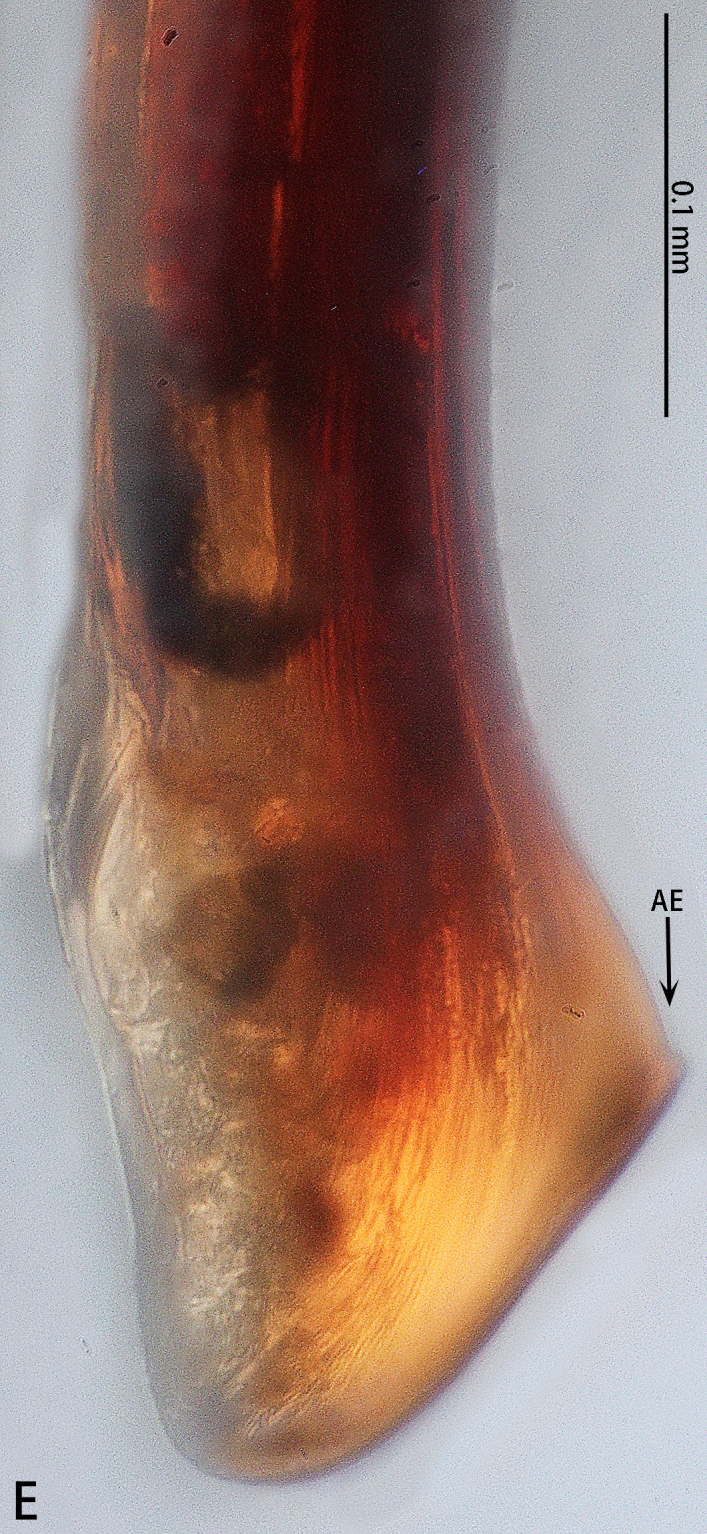


**Figure 6.**
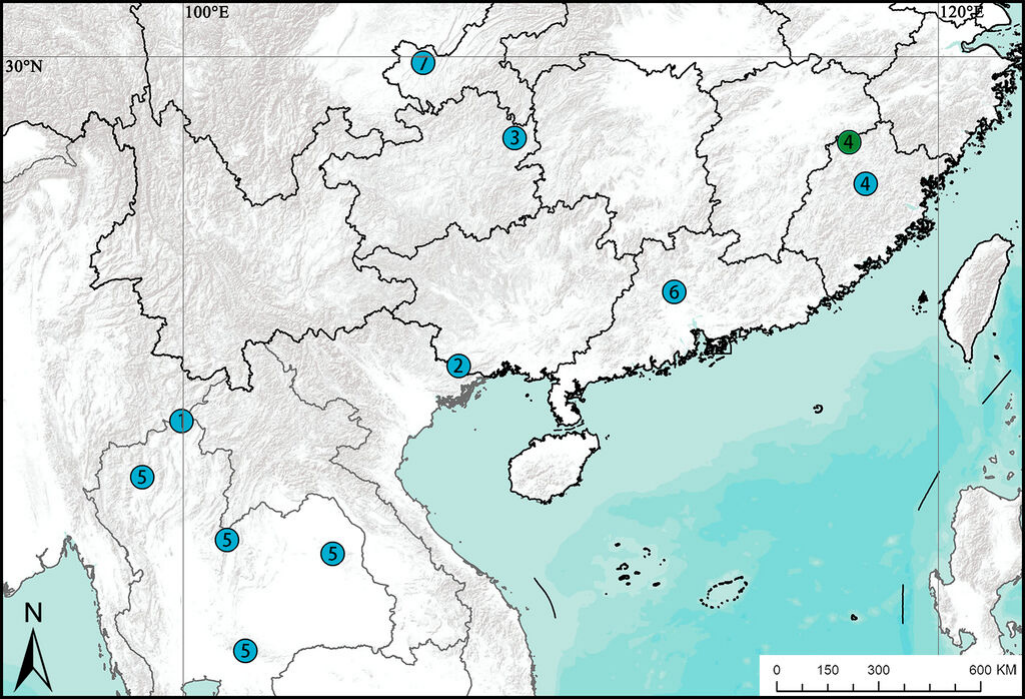
Distribution records of *Cyclocosmia* species (blue spots) and male *C.ricketti* in this study (green spot) in Asia: **1** *C.lannaensis* Schwendinger, 2005; **2** *C.latusicosta* Zhu, Zhang & Zhang, 2006; **3** *C.liui* Xu, Xu & Li, 2017; **4** *C.ricketti* (Pocock, 1901); **5** *C.siamensis* Schwendinger, 2005; **6** *C.sublatusicosta* Yu & Zhang, 2018; **7** *C.subricketti* Yu & Zhang, 2018.
